# Changes in Fish Assemblages following the Establishment of a Network of No-Take Marine Reserves and Partially-Protected Areas

**DOI:** 10.1371/journal.pone.0085825

**Published:** 2014-01-15

**Authors:** Brendan P. Kelaher, Melinda A. Coleman, Allison Broad, Matthew J. Rees, Alan Jordan, Andrew R. Davis

**Affiliations:** 1 New South Wales Fisheries, Department of Primary Industries, PO Box 4321, Coffs Harbour 2450, New South Wales, Australia; 2 National Marine Science Centre & Centre for Coastal Biogeochemistry Research, School of Environment, Science and Engineering, Southern Cross University, Coffs Harbour, New South Wales, Australia; 3 Institute for Conservation Biology & Environmental Management, School of Biological Sciences, University of Wollongong, New South Wales, Australia; CSIR- National institute of Oceanography, India

## Abstract

Networks of no-take marine reserves and partially-protected areas (with limited fishing) are being increasingly promoted as a means of conserving biodiversity. We examined changes in fish assemblages across a network of marine reserves and two different types of partially-protected areas within a marine park over the first 5 years of its establishment. We used Baited Remote Underwater Video (BRUV) to quantify fish communities on rocky reefs at 20–40 m depth between 2008–2011. Each year, we sampled 12 sites in 6 no-take marine reserves and 12 sites in two types of partially-protected areas with contrasting levels of protection (n = 4 BRUV stations per site). Fish abundances were 38% greater across the network of marine reserves compared to the partially-protected areas, although not all individual reserves performed equally. Compliance actions were positively associated with marine reserve responses, while reserve size had no apparent relationship with reserve performance after 5 years. The richness and abundance of fishes did not consistently differ between the two types of partially-protected areas. There was, therefore, no evidence that the more regulated partially-protected areas had additional conservation benefits for reef fish assemblages. Overall, our results demonstrate conservation benefits to fish assemblages from a newly established network of temperate marine reserves. They also show that ecological monitoring can contribute to adaptive management of newly established marine reserve networks, but the extent of this contribution is limited by the rate of change in marine communities in response to protection.

## Introduction

Human activities, such as catchment development, overfishing, pollution and maritime industries, have degraded marine and estuarine environments [1], [2]. Global concern for the health of marine systems has driven an unprecedented increase in marine protected area establishment over the last decade [3]. A small percentage of these marine protected areas are marine reserves where extraction of living marine resources is not permitted [4]. Many published studies have evaluated the responses of marine ecosystems to reserve establishment [5]. These include highlighting the types of species that do and do not benefit (e.g. [6], [7]) cascading trophic responses (e.g. [8], [9]), their influence on surrounding areas (e.g. [10], [11]), their influence on invasive species (e.g. [12]) and the enforcement effort required for significant changes to occur [13], [14].

While individual marine reserves provide conservation benefits, social and economic considerations often limit their size to a fraction of the bioregion whose biodiversity they are often designed to represent [4]. A limitation of most marine reserves is that they are not large enough to be completely self-sustaining because their size is less than the average dispersal distance of key species [15]. Although this issue can be resolved by establishing much larger marine reserves, socio-economic pressures are likely to prevent this, particularly on densely populated coasts. In an attempt to scale up the benefits of individual marine reserves to broader regions, networks of marine reserves are increasingly being established [4], [16]. Effective networks of marine reserves require adequate connectivity, such that each reserve can contribute and receive sufficient adults and larvae from adjacent reserves [4], [17]. Theoretical models suggest that a network of marine reserves may synergistically increase conservation benefits relative to the sum of the benefits from unconnected individual reserves [15], [18]–[20]. However, published data on changes in marine communities across marine reserve networks is limited relative to research on individual marine reserves and rigorous empirical tests of theoretical models optimizing marine reserve network designs are still in their infancy [4], [21].

Partially-protected areas are typically marine protected areas with less restrictive regulations than marine reserves [22], [23]. Depending on local objectives, they usually involve restrictions on particular activities, gear types, user groups, target species, or extraction periods [23]. Partially-protected areas may also be used to limit foreshore developments that require marine infrastructure (e.g. marinas or discharge outlets) thereby further reducing environmental threats [24]. Relative to marine reserves, there is much less published information about ecological changes associated with the establishment of partially-protected areas [23]. A meta-analysis of 20 studies found that partially-protected areas maintain higher biomasses, density and richness of marine organisms than areas with less regulation, but do not provide the same level of ecological benefits as no-take marine reserves [23]. These conclusions are, however, limited by (i) major differences in fishing restrictions in partially-protected areas among the different studies and (ii) most comparisons within a region being based on a single marine reserve or partially-protected area (but see [25]).

The establishment of multiple-use marine parks with replicated, closely spaced marine reserves, partially-protected areas and open access areas provides the opportunity to test hypotheses about networks of marine reserves and make rigorous comparisons of change in marine communities associated with different levels of environmental protection [26]. Over the last decade, six such multi-zoned marine parks containing 115 individual marine reserves (i.e. no-take sanctuary zones) have been established in the state waters of New South Wales, Australia [27]. Built into the legislation administering these marine parks are statutory requirements to review and, if necessary, adaptively manage the zoning arrangements 5 years after establishment. Some species can display significant changes after only a few years of protection (e.g. <5 years [28]), while others may take decades [29], [30]. Significant changes in marine community structure may take well in excess of 15 years [28], [31]. It is uncertain, therefore, the extent to which marine environmental monitoring will contribute to evidence-based adaptive management of marine park zoning arrangements at a 5 year review.

To assess the recovery trajectory of a newly established marine reserve network, we tested the hypothesis that reef-associated fish assemblages in reserves will change significantly relative to fished areas within 5 years of establishment. Concurrently, we also tested the hypothesis that reef-associated fish assemblages vary with different levels of environmental protection by including partially-protected areas. We also evaluated the performance of individual reserves within the network and related this to reserve size and enforcement actions.

## Materials and Methods

### Study Area

This study was undertaken in the Batemans Marine Park, a ∼85000 Ha multi-use marine park on the NSW South Coast, Australia (northern boundary = 35°31.086’S and southern boundary = 36°22.290’S) encompassing waters from the mean high tide mark to the limit of state waters (ca. 3 nm from land). The zoning plan for the marine park commenced in June 2007, after which activities (e.g. fishing, recreation, foreshore development, boating, pollution discharge, etc.) were regulated by the NSW Marine Parks Act (1998) and Regulations (1999, 2009), as well as a range of other legislation (e.g. Fisheries Management Act 1994, Coastal Protection Act 1979, Protection of Environmental Operations Act 1997, Threatened Species Conservation Act 1995, etc.). Marine park legislation specifically prohibits dredge and demersal trawling, mining and long-lining throughout the entire park.

As part of the objective to achieve conservation of biodiversity, the Marine Park was zoned into 4 types of areas: sanctuary zones, habitat protection zones, general use zones and special purpose zones, which represented 19.1%, 43.3%, 37.2% and 0.4% of the entire park, respectively. The different zone types are interspersed throughout the marine park creating a network of marine reserves and partially-protected areas. Special purpose zones were not included in the hypotheses tested because they only represented <0.5% of the marine park and were created for a range of specific management purposes (e.g. oyster farming, foreshore development and cultural resource use).

Sanctuary zones are strict no-take marine reserves that allow for non-extractive activities. Habitat protection zones are partially-protected areas where the species that can be harvested and the fishing methods that can be used are prescribed by legislation. For example, lawful recreational fishing methods are allowed in habitat protection zones with a few exceptions, but commercial purse seining, lift netting, mesh-netting, estuary prawn and haul netting are not permitted. With only the overall park-wide prohibitions enforced, general use zones are the least restrictive partially protected areas in NSW Marine Parks. Lawful commercial and recreational fishing methods other than trawling and long-lining are permitted in general use zones in the Batemans Marine Park. More specific details about prohibited activities can be found in Read and West [24].

### Sampling Methodology

Baited Remote Underwater Video (BRUV) was used to test hypotheses about changes in fish assemblages across the network of marine reserves and partially-protected areas. In many situations, BRUV units are preferred over other sampling techniques because they can be deployed in environments unsuitable for conventional diver based assessments [32], they are able to detect diver-shy species [33], they provide usable estimates of the relative abundance of economically-important species [33] and they provide a permanent visual record of surveys [32]. BRUV was particularly suitable for our study because it is a non-destructive sampling technique appropriate for high conservation areas (e.g. no-take marine sanctuaries) and survey depths often exceeded 30 m. Like all fish survey methods, BRUV only samples a subset of the fish community with a tendency towards sampling more predatory species than other methods on shallow reefs (e.g. underwater visual census [34]). The observed fish assemblage with BRUV systems can also be influenced by the presence of large predatory species (e.g. sharks [35]). Importantly, these issues did not systematically vary among zone types and, as such, did not influence the hypotheses that were tested here.

**Figure 1:**
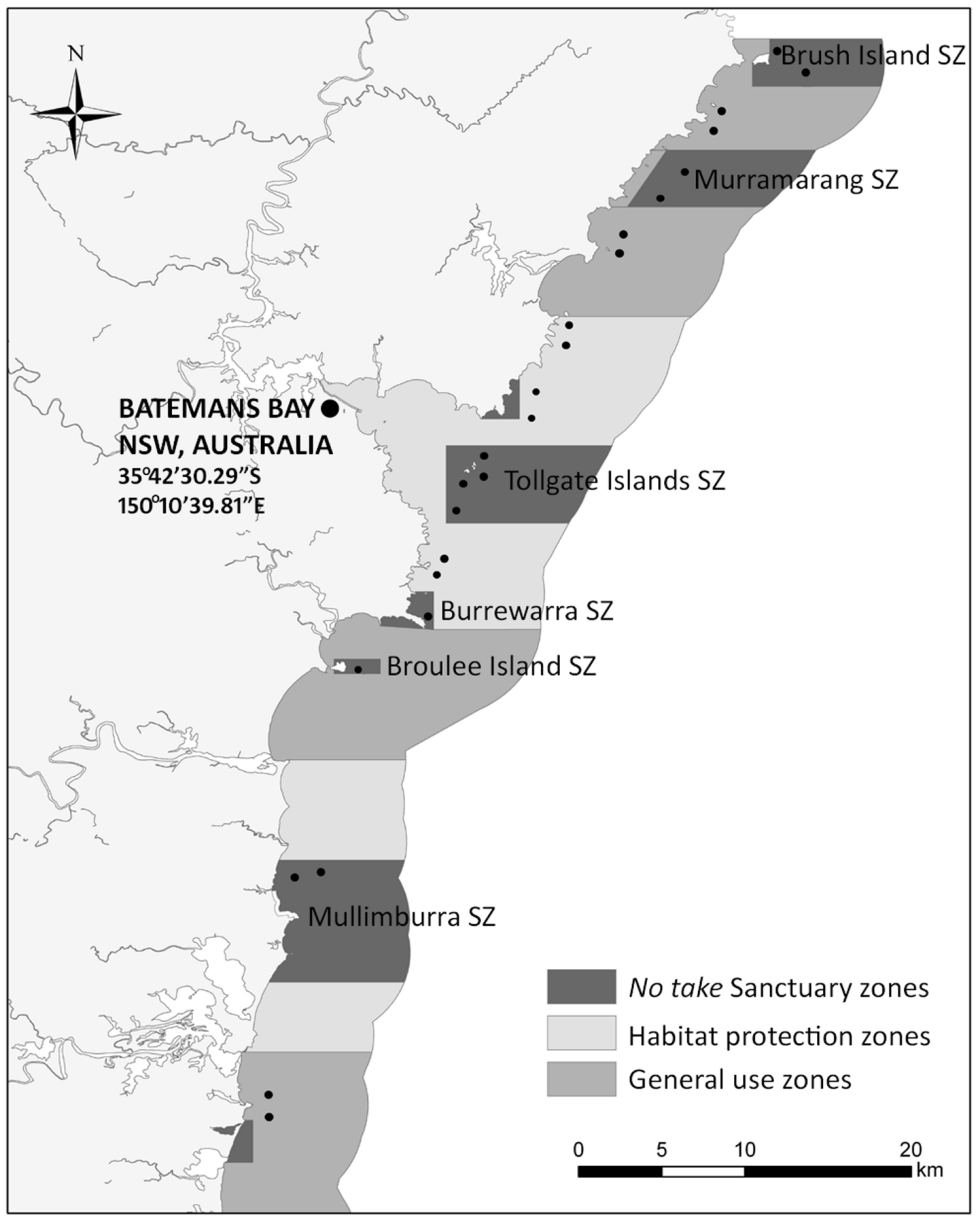
Map showing the configuration of zones in the part of the Batemans Marine Park (NSW, Australia) included in our study. The map highlights spatial arrangement of the network of no-take marine sanctuaries. • indicates the location of each BRUV sites.

BRUV units were deployed on rocky reef at 12 sites in sanctuary zones, 6 habitat protection and 6 general use zones (Fig. 1). This design allowed for planned balanced comparisons between no-take and fished areas (12 sites vs 12 sites) and between the two types of partially protected areas (6 sites vs 6 sites) (see design below). The sanctuary zones included were between 2 km to 14 km apart, which is likely within the range of either larval or adult movements for many common reef fish species (e.g. [36] and references within), especially considering the East Australia Current [37], [38]. Sites were haphazardly interspersed throughout the Marine Park from Brush Island (35°31.086’S) to Potato Point (35°06.172’S) (Fig. 1). Each site was dominated by rocky reef and was sampled in 2008, 2009, 2010 and 2011. In 2008, sampling occurred from January to May and for the following years it occurred from June to August. This change in timing was related to the implementation of a state-wide monitoring program and was not a major consideration for interpretation of results because there is often no clear seasonal signal in demersal fish assemblages in this region [39]. This likely stems from substantial spatial-temporal variation and relatively mild winters. Moreover, the key hypotheses of this study focused on differences between sanctuary zones and partially protected areas rather than temporal variation.

In each site, 4 BRUV units were deployed at approximately 200 m intervals onto reef habitat. The mean (±S.E.) depth of deployments was 26.0 (1.3) m, 26.3 (1.6) m and 26.6 (0.5) m for sanctuary, habitat protection and general use zones, respectively, and did not differ significantly among zones (PERMANOVA, *pseudo-F*_2,21_ = 1.48, *P* = 0.13). Each BRUV unit was constructed as per Malcolm et al. [40], which included a galvanized metal frame containing a video camera (mini DV SONY) pointed at a bait bag mounted horizontally at the end of a 1.5 m long bait arm. Cameras were housed within high-pressure polyvinyl chloride pipe with flat acrylic end-ports yielding a field of view of 110°. For each BRUV deployment, the bait bag was replenished with ∼500 g of chopped pilchards (*Sardinops* spp.) and each BRUV unit was left on the bottom for 30 minutes. This bait type was determined to yield the most consistent outcomes compared to others previously tested (e.g. abalone viscera or crushed urchin [41]). This bottom time was considered appropriate for sampling reef fish between 20–40 m because there is no significant differences among fish assemblages and the *max* N of many common species when deployment times of 30, 60, and 90 minutes were compared (D. Harasti, unpublished data). Furthermore, the replication levels of sites and camera deployments within sites provide adequate power to reliably detect significant differences between fish species richness and total *max* N in sanctuary zones compared to fished areas with mean differences of 30% and 100%, respectively (B. Kelaher, unpublished data).

Videos were analyzed in the laboratory using a field of view 2 m behind the bait bag, which represented a standardized area of 9.4 m^3^ [40]. For each replicate BRUV deployment, we determined species richness, total *max* N, and *max* N of each fish species. *Max* N for a species was the maximum number of individuals in any frame and total *max* N was the sum of *max* N’s for each deployment [32]. When the abundances of families of fishes were analyzed, the *max* N value used for each replicate was the summed *max* N of each fish species in that family. Analyses were restricted to fin fish to avoid complications associated with extra protection of all but two species of elasmobranchs (i.e. *Mustelus antarcticus* and *Galeorhinus galeus*) in habitat protection zones.

### Comparisons across a Network of Marine Reserves and Partially-protected Areas

Hypotheses about changes in fish assemblages across the network of no-take marine reserves and partially-protected areas were tested using 2 factor analyses with *zone type* (3 levels, orthogonal and fixed) and *years* since the commencement of the zoning plan (4 levels, orthogonal and fixed), with analyses based on site averages. To test for differences in fish assemblages between no-take sanctuary zones and fished areas a contrast was included to compare sanctuary zones against zones where fishing was allowed. To test for differences in fish assemblages between the two types of partially-protected zones a contrast was included comparing fish assemblages in habitat protection and general use zones.

Hypotheses were based on multivariate comparisons of fish assemblage structure and univariate comparisons of fish species richness and total *max* N. Hypotheses were also tested using the total *max* N of four numerically-dominant families, Carangidae, Kyphosidae, Labridae and Monacanthidae, which represented 17%, 29%, 14% and 7% of the overall total *max* N, respectively. Analyses were also conducted on fish species with a summed *max* N that totaled more than 300 individuals and are commonly-caught in NSW waters. Each species is currently assessed as either moderately fished, fully fished, growth overfished or overfished indicating that they are each under fishing pressure. These taxa were *Pagrus auratus* [snapper, growth overfished], *Pseudocaranx georgianus* [silver trevally, growth overfished], *Scorpis lineolata* [silver sweep, moderately to fully fished], *Ophthalmolepis lineolatus* [southern maori wrasse, moderately fished], *Trachurus novaezelandiae* [yellow tail scad, fully fished] and *Nemadactylus douglasii* [grey morwong, overfished] (see [42] for details). In Batemans Marine Park each of the above species is caught recreationally, as well as in the commercial ocean trap and line fishery. However, *T. novaezelandiae* is mostly caught in purse seine nets [42], which cannot be used in habitat protection zones.

Hypotheses about changes in fish assemblages and individual families and species were tested with non-parametric multivariate analysis of variance (PERMANOVA [43]). These non-parametric procedures are robust to variable ecological data commonly obtained from marine communities [44]. All univariate analyses were done using Euclidean distance to create similarity matrices. All multivariate analyses used the Bray-Curtis similarity coefficient [45]. Non-metric multidimensional scaling (nMDS) [46] was used to generate two-dimensional ordination plots which graphically illustrated multivariate patterns in fish assemblages.

### Comparisons of the Performance of Individual Marine Reserves

The 12 sanctuary zone sites were located within six of the 10 offshore sanctuary zones in the Batemans Marine Park. From north to south, these were Brush Island, Murramarang, Tollgate Islands, Burrewarra Point, Broulee Island and Mullimburra (GPS co-ordinates of boundaries included in the NSW Marine Parks (Zoning Plan) Regulation 1999). These zones encompassed the smallest and largest offshore sanctuary zones in the marine park. As well as size, these sanctuary zones varied across a range of marine park planning criteria (see Table 1 for details). To compare the individual performance of these 6 sanctuary zones since the commencement of the zoning plan, a ratio was established with (*x*_SZ_+1)/(*X*_FA_+1), where *x*_SZ_ was the response variable from each sanctuary zone BRUV deployment and *X*_FA_ was the average of the closest two sites in areas where fish could be legally caught. This sanctuary zone/fished area ratio (hereafter called SZ/FA ratio) provided an indication of relative changes in fish assemblages in no-take and fished zones in a local area around individual sanctuary zones rather than across the network of reserves and partially-protected areas.

**Table 1:** Average direction of association for the six abundant and commonly-caught fish species (see methods) since the commencement of the zoning plan and potential explanatory variables in six offshore sanctuary zones in the Bateman Marine Park.

	Brush Island	Murramarang	Tollgate Islands	Burrewarra	Broulee Island	Mullimburra	*r*	*P*
**Direction of association since the establishment of the zoning plan**								
Average Pearson’s *r* for fish response variables	−0.07	−0.46	0.64	0.06	−0.19	−0.20		
**Potential explanatory variables**								
Enforcement actions	8	7	104	11	8	26	0.91	**<0.01**
Sanctuary zone area (ha)	1709	2449	3291	312	172	4542	0.12	0.82
Terminates at 3 nm boundary	Yes	Yes	Yes	No	No	Yes		
Directly Linked to estuarine sanctuary zones	No	No	No	No	No	Yes		
Buffered by Habitat Protection Zone	No	No	Yes	Part	No	Yes		
Adjacent to mainland National Park/Nature reserve	Yes	Yes	No	No	Yes	Yes		

To test whether the performance of individual sanctuary zones was variable, a two factor PERMANOVA analysis was carried out on overall fish species richness, total *max* N and the total *max* N of four numerically-dominant families: Carangidae, Kyphosidae, Labridae and Monacanthidae with the factors *sanctuary zones* (*SZ*, 6 levels orthogonal and random) and *year*s since zoning plan commencement (4 levels, orthogonal and fixed). These univariate analyses used Euclidean distance to create similarity matrices and were based on individual BRUV deployments.

The average direction of change of the six key fish outlined above (*Pagrus auratus*, *Pseudocaranx georgianus*, *Scorpis lineolata*, *Ophthalmolepis lineolatus*, *Trachurus novaezelandiae* and *Nemadactylus douglasii*) in each sanctuary zone was determined by calculating Pearson’s correlation coefficient for the average SZ/FA ratio vs years since commencement of the zoning plan. These correlation coefficients were then averaged to determine a generalized direction of change (*r*_av_) for each individual sanctuary zone with *r*_av_ being a value between −1 and 1 with positive and negative values indicative of positive and negative associations between SZ/FA ratio and time since establishment, respectively. To evaluate potential explanations for variation in individual sanctuary zone performance, the *r*_av_ for individual sanctuary zones were correlated using Pearson’s correlation coefficient with the number of enforcement actions by marine park staff from 1 July 2009 to 30 June 2011 and the size of the sanctuary zone. To control for Type 1 error, the significance level of these correlations was corrected with sequential Bonferroni’s technique [47]. Qualitative comparisons were also made between individual sanctuary zone performance and other important aspects of individual sanctuary zones, including whether they (i) terminated at the 3 nm limit maximizing cross shelf diversity, (ii) were directly linked to no-take estuarine areas facilitating connectivity, (iii) were buffered by habitat protection zones limiting accidental damaging activities or (iv) were adjacent to terrestrial reserves reducing land-based impacts (e.g. urban run-off).

### Ethics Statement

This study was conducted with the permission of the NSW Marine Parks Authority and the NSW Department of Primary Industries. BRUV work was done under the auspices of the University of Wollongong animal ethics committee (approval number AE12/07). The study complied with the current laws of Australia.

## Results

### Comparisons across a Network of Marine Reserves and Partially-protected Areas

**Figure 2:**
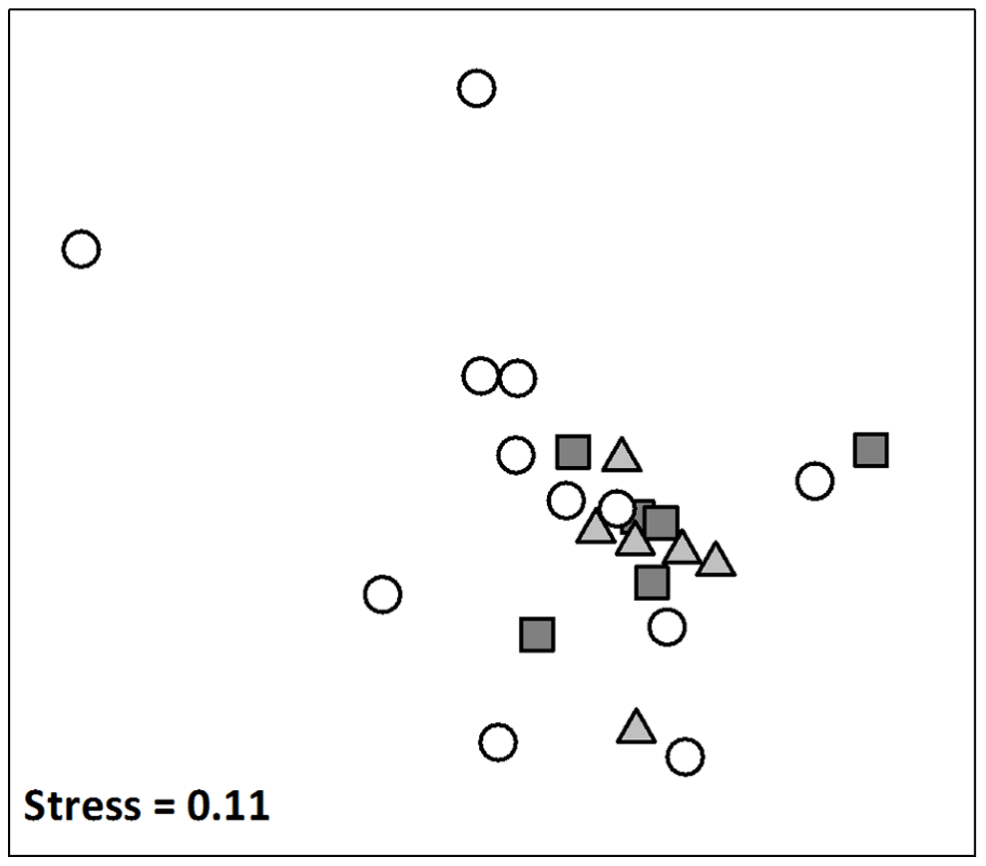
nMDS ordination of fish assemblages represented as centroids for each site within sanctuary (white circles), habitat protection (light grey triangles) and general use (dark grey squares) zones since the commencement of the zoning plan for the Batemans Marine Park. As there were no significant interactions between years and main effect contrasts (Table 1), points indicate site centroids averaged across years since establishment.

In total, 17,681 individuals from 89 species of fin fish were identified from the 384 BRUV deployments from 2008–2011. The structure of fish assemblages in no-take marine reserves (i.e sanctuary zones) differed significantly from fished areas (Table 2, Fig. 2). In contrast, the structure of fish assemblages in habitat protection zones did not differ significantly from general use zones (Table 2, Fig. 2).

**Table 2:** PERMANOVA analyses comparing the structure of fish assemblages using multivariate data on the richness and abundance (total *max* N) of fishes among zone types and among years since the commencement of the zoning plan.

		(a) Fish assemblages			(b) Species richness			(c) Total *max* N		
	df	MS	*p-F*	*P*	MS	*p-F*	*P*	MS	*p-F*	*P*
Zone type	2	3926.20	2.16	**<0.01**	21.17	2.34	0.10	3210.50	5.61	**<0.01**
SZ vs FA	1	5352.00	2.96	**<0.01**	5.16	0.56	0.48	5188.40	9.22	**<0.01**
HPZ vs GUZ	1	2500.40	1.41	0.17	37.19	7.57	**<0.01**	1232.70	4.42	0.05
Years	3	4194.00	2.30	**<0.01**	47.52	5.26	**<0.01**	1978.90	3.46	**<0.05**
Zone type × Years	6	1058.90	0.58	0.99	3.86	0.43	0.86	228.44	0.40	0.88
Years × SZ vs FA	3	894.56	0.49	0.99	4.73	0.52	0.67	363.55	0.65	0.60
Years × HPZ vs GUZ	3	1223.20	0.69	0.92	2.98	0.61	0.60	93.32	0.33	0.79
Residual	84	1820.70			9.03			571.82		

Fish assemblages (a) used Bray-Curtis similarity measures following square root transformation while species richness (b) and total *max* N (c) used Euclidean distance to generate similarity matrices. Contrasts were included to compare sanctuary zones (SZ) with fished areas (FA) and habitat protection zones (HPZ) with general use zones (GUZ).

*p-F* = pseudo *F* ratio generated by PERMANOVA.

**Figure 3:**
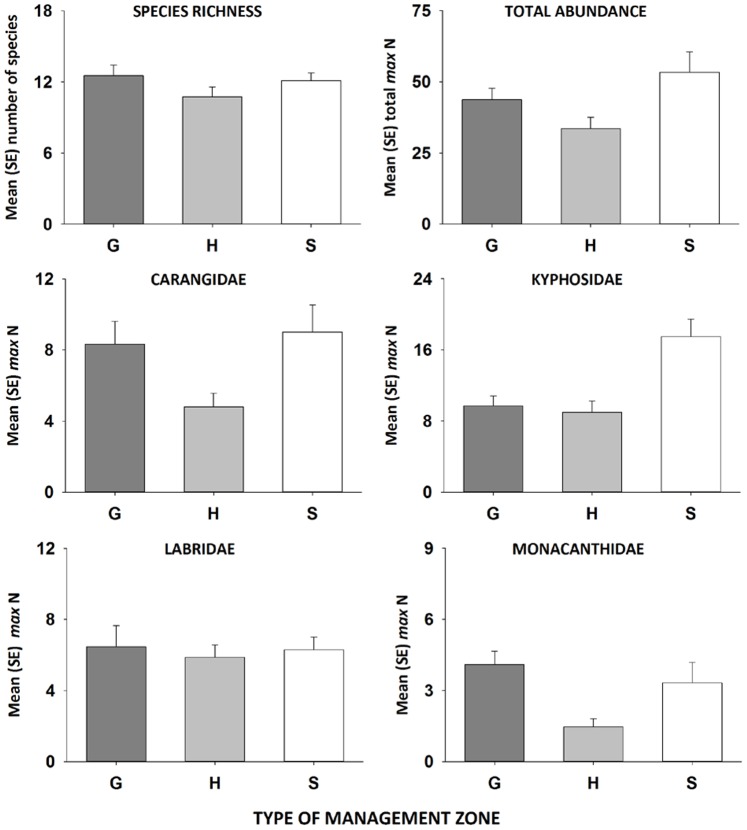
Mean (±1 SE) richness and total *max* N of fish assemblages and numerically-dominant family groups in general use (dark grey bars), habitat protection (light grey bars) and sanctuary (white bars) zones since the commencement of the zoning plan for the Batemans Marine Park.

The richness of fish species was significantly greater in general use zones than in habitat protection zones, but did not differ significantly between sanctuary zones and fished areas (Table 2, Fig. 3). In contrast, the total *max* N of fishes was 37% greater in no-take marine reserves (i.e. sanctuary zones) compared to fished areas, which was significant (Table 2, Fig. 2). There was a trend towards more fish in general use zones compared to habitat protection zones (*P* = 0.053, Fig. 3). Of the numerically-dominant families examined, the mean *max N* of kyphosids was significantly higher in no-take sanctuary zones compared to fished areas (Table 3, Fig. 3). For monacanthids, however, the differences in mean *max* N were significant between fished zones (Table 3, Fig. 3). The total *max* N of carangids and labrids did not differ significantly among zone types (Table 3).

**Table 3:** PERMANOVA analyses comparing the total *max* N of the numerically-dominant families among zone types and among years since the commencement of the zoning plan using Euclidean distance.

		(a) Carangidae			(b) Kyphosidae			
	df	MS	*p-F*	*P*	MS	*p-F*		*P*
Zone type	2	146.12	1.32	0.29	803.43	6.03		**<0.01**
SZ vs FA	1	142.59	1.32	0.26	1600.70	12.37		**<0.01**
HPZ vs GUZ	1	149.64	2.11	0.17	6.20	0.14		0.70
Years	3	92.76	0.84	0.50	48.76	0.37		0.77
Zone type × Years	6	19.05	0.17	0.98	72.47	0.54		0.78
Years × SZ vs FA	3	15.91	0.15	0.93	81.74	0.63		0.58
Years × HPZ vs GUZ	3	22.19	0.31	0.84	63.20	1.38		0.26
Residual	84	110.57			133.24			
		**(c) Labridae**			**(d) Monacanthidae**			
	**df**	**MS**	***p-F***	***P***	**MS**	***p-F***	***P***	
Zone type	2	2.27	0.42	0.65	44.87	5.12	**<0.01**	
SZ vs FA	1	0.32	0.06	0.82	7.04	0.74	0.40	
HPZ vs GUZ	1	4.23	0.99	0.32	82.69	9.94	**<0.01**	
Years	3	63.84	11.71	**<0.01**	15.89	1.82	0.15	
Zone type × Years	6	2.18	0.40	0.89	9.64	1.10	0.37	
Years × SZ vs FA	3	1.14	0.21	0.88	12.15	1.27	0.31	
Years × HPZ vs GUZ	3	3.21	0.75	0.53	7.14	0.86	0.46	
Residual	84	5.45			8.76			

Contrasts were included to compare sanctuary zones (SZ) with fished areas (FA) and habitat protection zones (HPZ) with general use zones (GUZ).

*p-F* = pseudo *F* ratio generated by PERMANOVA.

**Figure 4:**
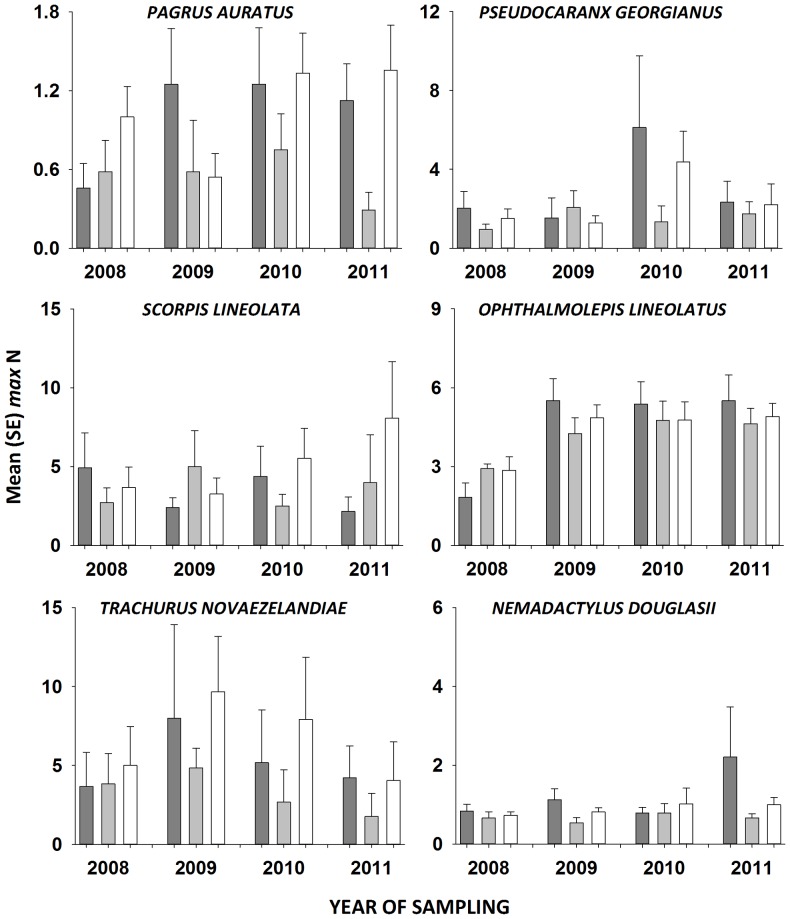
Mean (±1 SE) *max* N of fish species important to recreational and commercial fisheries in general use (dark grey bars), habitat protection (light grey bars) and sanctuary (white bars) zones in each year since the commencement of the zoning plan for the Batemans Marine Park.

In general, the *max* N’s of individual species were more variable than univariate community measures (i.e. species richness and total *max* N) or family groups, leading to fewer significant results. There was, however, a trend towards more *P. auratus* (snapper) in sanctuary zones than fished areas in 2008, 2010 and 2011 (Fig. 4). The *max* N of *P. georgianus* (silver trevally), *S. lineolata* (silver sweep), *O. lineolatus* (southern maori wrasse), *T. novaezelandiae* (yellow tail scad) and *N. douglasii* (grey morwong) did not vary significantly among zone types since the zoning plan’s establishment (Table 4, Fig. 4). The average *max* N of *S. lineolata* increased in sanctuary zones with years since establishment.

**Table 4:** PERMANOVA analyses comparing the *max* N of key fish species among zone types and among years since the commencement of the zoning plan.

		(a) *P. auratus*			(b) *P. georgianus*			(c) *S. lineolata*		
	df	MS	*p-F*	*P*	MS	*p-F*	*P*	MS	*p-F*	*P*
Zone type	2	2.20	2.95	0.07	13.20	1.06	0.37	31.73	0.81	0.45
SZ vs FA	1	1.76	2.32	0.15	0.15	0.01	0.92	63.38	1.67	0.22
HPZ vs GUZ	1	2.64	4.51	**<0.05**	26.26	1.96	0.18	0.08	0.01	0.95
Years	3	0.74	0.99	0.41	27.44	2.20	0.09	5.88	0.15	0.94
Zone type × Years	6	0.87	1.16	0.34	8.70	0.70	0.70	28.19	0.72	0.62
Years × SZ vs FA	3	1.21	1.59	0.19	1.38	0.11	0.95	37.98	1.00	0.41
Years × HPZ vs GUZ	3	0.53	0.90	0.44	16.02	1.19	0.31	18.40	0.96	0.44
Residual	84	0.75			12.48			39.20		
		**(d) ** ***O. lineolatus***			**(e) ** ***T. novaezelandiae***			**(f) ** ***N. douglasii***		
	**df**	**MS**	***p-F***	***P***	**MS**	***p-F***	***P***	**MS**	***p-F***	***P***
Zone type	2	1.04	0.31	0.74	92.33	1.07	0.36	1.86	1.87	0.13
SZ vs FA	1	<0.01	<0.01	1.00	137.16	1.65	0.19	0.07	0.06	0.85
HPZ vs GUZ	1	2.08	0.70	0.43	47.50	0.95	0.38	3.66	2.64	0.07
Years	3	31.49	9.37	**<0.01**	70.34	0.81	0.48	1.33	1.34	0.27
Zone type × Years	6	1.94	0.58	0.76	9.65	0.11	0.99	1.02	1.03	0.39
Years × SZ vs FA	3	0.69	0.21	0.90	12.79	0.15	0.93	0.51	0.49	0.77
Years × HPZ vs GUZ	3	3.20	1.08	0.40	6.52	0.13	0.95	1.53	1.11	0.33
Residual	84	3.36			86.55			0.99		

Contrasts were included to compare sanctuary zones (SZ) with fished areas (FA) and habitat protection zones (HPZ) with general use zones (GUZ).

*p-F* = pseudo *F* ratio generated by PERMANOVA.

### Comparisons of the Performance of Individual Marine Reserves

**Figure 5:**
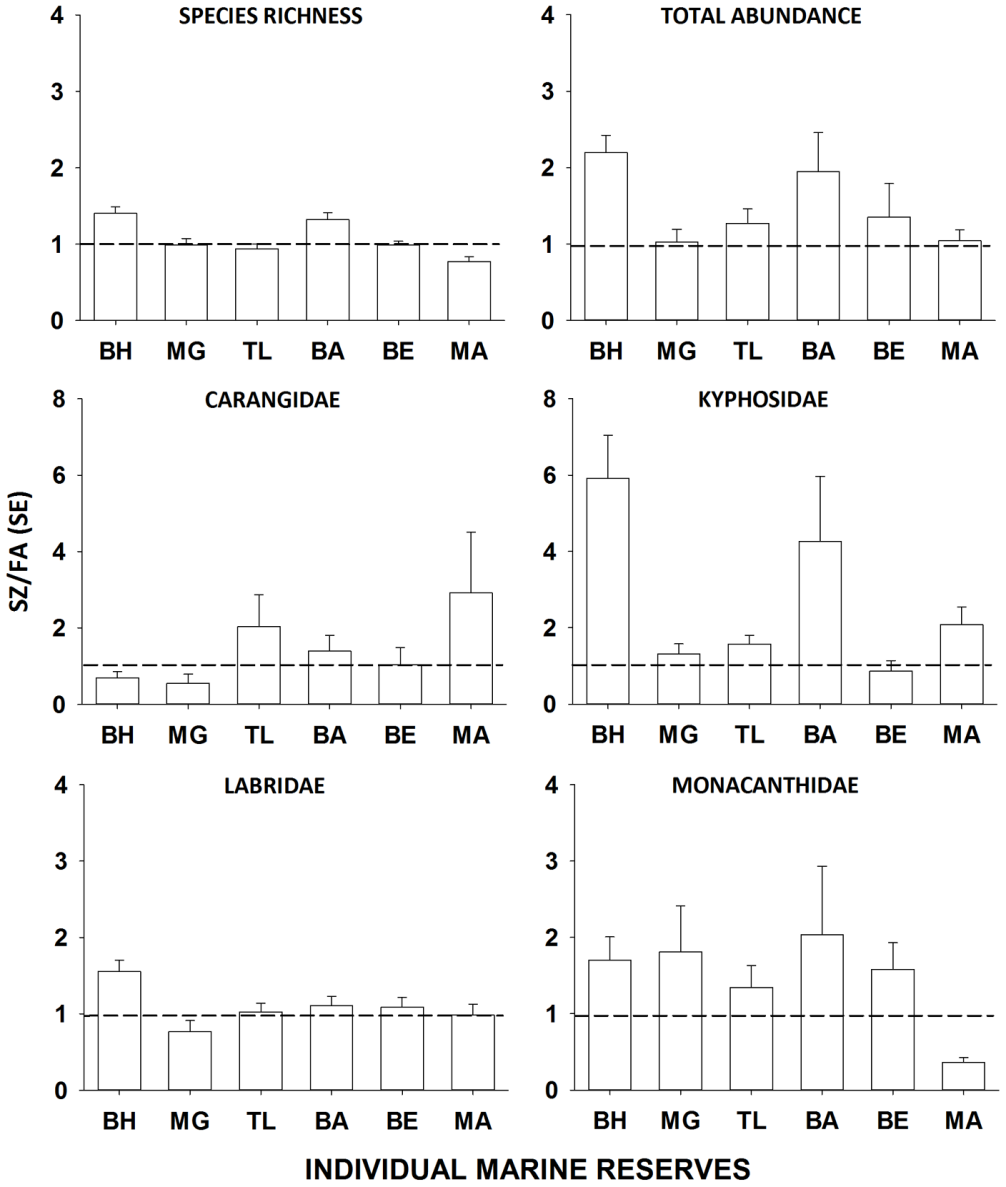
Mean (±1 SE) sanctuary zone to local fished areas ratio (SZ/FA) for richness and total *max* N of fish assemblages and numerically-dominant family groups in the Brush Island (BH), Murramarang (MG), Tollgate Islands (TL), Burrewarra (BA), Broulee Island (BE) and Mullimburra (MA) sanctuary zones. Bars represent the main effects of sanctuary zone averaged across years since establishment.

There were significant differences in the SZ/FA ratio in the richness and total *max* N of fish assemblages among individual sanctuary zones (Table 5, Fig. 5). These ratios indicated a trend for greater *max* N of fish in sanctuary zones (i.e. the probability (*P*) of 6 ratios greater than 1 = 0.059) and substantially richer fish assemblages in two of the six sanctuary zones sampled (i.e. where the mean plus standard error bar is greater than 1 on Fig. 4). However, the average number of fish species in the Mullimburra sanctuary zone plus one standard error was less than 1, indicating fewer fish species in this sanctuary zone relative to the surrounding fished area (i.e. mean plus standard error are less than 1 on Fig. 5).

**Table 5:** PERMANOVA analyses comparing the sanctuary zone to local fished area ratio (SZ/FA) for univariate measures of fish assemblages, numerically-dominant families and key fish species among sanctuary zones (SZ) and among years since the commencement of the zoning plan for the Batemans Marine Park.

		(a) Species richness			(b) Total abundance		
	df	MS	*p-F*	*P*	MS	*p-F*	*P*
SZ	5	1.70	16.15	**<0.01**	6.97	4.57	**<0.01**
Years	3	0.18	1.24	0.33	4.39	2.25	0.13
SZ × Years	15	0.15	1.47	0.13	2.03	1.33	0.21
Residual	168	0.11			1.52		
		**(c) Carangidae**			**(d) Kyphosidae**		
	**df**	**MS**	***p-F***	***P***	**MS**	***p-F***	***P***
SZ	5	26.72	3.38	**<0.05**	114.14	8.60	**<0.01**
Years	3	2.64	0.12	0.96	30.17	2.04	0.14
SZ × Years	15	25.65	3.24	**<0.01**	15.05	1.13	0.35
Residual	168	7.91			13.27		
		**(e) Labridae**			**(f) Monacanthidae**		
	**df**	**MS**	***p-F***	***P***	**MS**	***p-F***	***P***
SZ	5	2.18	6.77	**<0.01**	9.98	4.28	**<0.01**
Years	3	1.10	2.30	0.12	15.18	3.29	**<0.05**
SZ × Years	15	0.51	1.57	0.10	5.04	2.16	**<0.05**
Residual	168	0.32			2.33		

*p-F* = pseudo *F* ratio generated by PERMANOVA.

The SZ/FA ratio of total *max* N for carangids, kyphosids and labrids also varied significantly among individual sanctuary zones demonstrating variation in individual reserve performance (Table 5, Fig. 5). For four out of six sanctuary zones, the SZ/FA ratio was close to one. For labrids, however, the average total *max* N plus one standard error was less than 1 in the Murramarang reserve indicating fewer of these fishes in this sanctuary zone than in the surrounding fished areas (Fig. 4). The SZ/FA ratio of total *max* N for carangids and monacanthids interacted significantly among sanctuary zones and years since establishment (Table 5). Post hoc tests (PHT) revealed that the patterns of average SZ/FA ratio for these fish taxa varied significantly among sanctuary zones in some years but not others (PHT: *P*<0.05). For example, the SZ/FA ratio of carangids did not vary among individual sanctuary zones in 2008 and 2011, but was significantly greater at Mullimburra than other sanctuary zones in 2009 (PHT: *P*<0.05) and significantly smaller than other sanctuary zones in 2010 (PHT: *P*<0.05). The average SZ/FZ ratio for monacanthids and carangids indicated more of these fishes in 5 out of 6 and 3 out of 6 sanctuary zones than the surrounding fished areas, respectively (Fig. 4). The average SZ/FA ratio plus one standard error for monacanthids at the Mullimburra reserve was less than 1 for each year of sampling (2008 SZ/FA ratio [SE] = 0.41 [0.08], 2009 = 0.32 [0.04], 2010 = 0.21 [0.03], 2011 = 0.51 [0.085]). Similarly, the average SZ/FA ratio plus one standard error of carangids in the Murramarang reserve, was less than 1 for three of the four years of sampling (2008 SZ/FA ratio [SE] = 0.67 [0.10], 2009 = 0.28 [0.06], 2010 = 0.11 [0.02], 2011 = 1.17 [0.61]).

The *r*_av_ for the six abundant species considered to be important for commercial and recreational fishing in NSW waters varied substantially among individual sanctuary zones (Table 1). The value of 0.64 for the Tollgate Island sanctuary zone was strongly positive with each of the six species having a positive association between SZ/FA ratio and the years since the zoning plan’s commencement (Table 1). In contrast, the average direction of change at Murramarang (*r*_av_ = −0.46) and Mullimburra (*r*_av_ = −0.20) suggested limited performance in these marine reserves for the species we considered. For the remaining three sanctuary zones, there was no strong average directional association between SZ/FA ratio and the years since the zoning plan’s commencement (0.20>*r*_av_<−0.20).

After *P*-values were corrected using sequential Bonferroni’s technique, there was a significant correlation between the *r*_av_ for the six key fish species and the number of enforcement actions undertaken (Table 1), indicating a positive association between individual reserve performance and compliance activity. In contrast, there were no significant correlations between average directional association of the SZ/FA ratio for the six key fish species since the park’s establishment (*r*_av_) and the size of sanctuary zones (Table 1). With respect to key reserve attributes (Table 1), the large reserve at Mullimburra was the only one to have full coverage from the shore to the 3 nm limit of state waters, to be directly linked to estuarine sanctuary zones, to be buffered by habitat protection zones and to be adjacent to mainland National Parks. In contrast, the only key attribute possessed by the relatively small Broulee Island Reserve was that it was adjacent to a Nature Reserve. Moreover, the small Burrewarra reserve only had partial buffering from habitat protection zones. The reserves at Brush Island and Murramarang each terminated at the 3 nm limit and were adjacent to National Parks. The Tollgate Island Reserve ran out to the 3 nm limit of state waters and was buffered by habitat protection zones. As it commenced approximately 1 km offshore (Fig. 1), the Tollgate Island reserve could not link directly to estuarine sanctuary zones. It was, however, directly adjacent to the Clyde River Estuary that included several substantial no-take estuarine sanctuaries. Although the Tollgate Islands Reserve was adjacent to urban development, its distance from shore (Fig. 1) provided a substantial buffer from land-based impacts. The Tollgate Islands themselves are Nature Reserves not accessible to the general public.

## Discussion

On average there were 38% more fish in the network of marine reserves than in fished areas of the Batemans Marine Park. The largest contribution to this effect came from Kyphosids (drummers). Compared to global averages for individual reserves (e.g. 166%, n = 124 reserves [5]) the elevated fish abundances across the network of marine reserves was modest, but well within the spectrum of positive responses. This may, in part, be due to the marine park only being in place for 5 years (e.g. [30], [48]) and previous fishing pressure being regulated by conventional fisheries management [42]. It may also stem from the fished areas being partially-protected such that even the most unprotected places in the marine park (general use zones) were free from potentially damaging activities such as demersal trawling and long-lining [24].

Despite the total *max* N of fishes being significantly greater in marine reserves than in fished areas, there were no significant differences in the richness of reef fishes across the network of no-take reserves compared to fished areas. There was, however, greater richness of reef fish species in Brush Island and Burrewarra reserves compared to the adjacent fished areas. Similar to richness, there were also no significant differences in the abundances of some family groups and commonly-caught fish species among zone types. Large variation in the measurement of fish populations contributed to these results. For example, although there was 37% more Carangids in marine reserves than in fished areas, this comparison was not close to being significant due to substantial variation among zones and sites in fished areas. Nonetheless, it was to be expected that only the very large changes in fish assemblages would be detected because power analyses demonstrated that, for the levels of replication used, effects of 30% and 100% were required to reliably detect significant differences in the richness and max *N* between reserves and fished areas, respectively.

Another consideration for the non-significant results was the influence of time since reserve establishment. In comparisons of other temperate Australian marine reserves to fished areas from before to three years after establishment, Edgar et al [49] demonstrated few changes in the abundance of fish and invertebrates in the marine reserves compared to fished areas. They concluded that the three-year period studied after reserve commencement may have been insufficient to generate clear trends in fish population recoveries. The results from our study suggest that 5 years may also not be sufficient to detect change of some fish species whose abundances have been demonstrated to recover in much older marine reserves (e.g. *Pagrus auratus*, snapper [28]). Similar conclusions were reached about fish populations on shallow subtidal reefs sampled using underwater visual census over the first five years following the establishment of the Batemans Marine Park [50].

An important consideration for interpreting positive effects of marine reserves on fish abundances is whether marine reserves were deliberately placed in areas with more fish. For the Batemans Marine Park this was not the case because, although some data was collected prior to the parks’ establishment [31], [51], detailed regionally specific data on reef fish assemblages and reef extent and complexity were not available to marine park planners prior to the marine parks establishment. Furthermore, there are two lines of evidence to support positive reserve effects: (i) the abundances of some species increased in sanctuary zones over time (e.g. *S. lineolata,* silver sweep) and (ii) sampling conducted prior to the zoning plan’s establishment indicated that there were similar if not fewer fish in marine reserves compared to fished areas [48].

Given that all levels in the factor *zone type* were replicated with multiple sites and BRUV deployments were haphazardly located on reefs of similar structure, our study was of the form of a standard ecological field experiment where the manipulation was the implementation and enforcement of marine park regulations. While this sampling was sufficient for testing the proposed hypotheses about reserve effects, similar to most published field experiments, the ability to attribute treatment effects to the manipulation (e.g. conservation measures in this case) rather than site selection could be improved by the incorporation of pre-establishment data into comparisons (e.g. BACI-type experimental designs [52]). The marine park planning process is, however, not always conducive to implementation of robust BACI experimental designs. For the Batemans Marine Park, there was around 14 months between the declaration of the Park and the implementation of the zoning plan [27]. Most of this period was taken up with planning and public consultation, leaving only a few months between when the locations of the marine reserves were finalized and the zoning plan coming into effect. Consequently, there was insufficient time to collect the inter-annual pre-establishment data required for a temporally-replicated BACI-style experimental design.

In general, fish assemblages either did not differ between the partially-protected areas with different levels of protection or there were more species in general use zones than habitat protection zones. There was, therefore, no evidence that the additional restrictions associated with habitat protection zones, such as removal of commercial purse seining, lift netting and set lining, improved conservation outcomes for reef fish assemblages on offshore reefs after 5 years. Given that trawling and long-lining were removed from the entire Batemans Marine Park, the removal of other less damaging commercial fishing activities from habitat protection zones probably had limited additional influence on fish communities. Furthermore, the designation of habitat protection zones could have attracted some increased recreational fishing effort, therefore reducing differences between the two different zones. This is because habitat protection zones are often promoted as enhancing recreational fishing opportunities through reduced commercial fishing effort, with similar types of areas in NSW estuaries (e.g. Recreational Fishing Havens) being perceived by recreational anglers as improving catch rates [53]. Management strategies that result in shifting recreational fishing effort towards partially-protected areas may limit the conservation benefits of these areas.

Although there was a general increase in overall fish abundance in marine reserves across the network, there was significant variation among the performance of individual reserves. The six commonly caught fish species in marine reserves at the Tollgate Islands showed the strongest positive trend over the 5 years of reserve protection (Pearson’s *r* = 0.64). Although quantitative data on fishing effort was not collected consistently across the Batemans Marine Park prior to its establishment, it was well known that the Tollgate Islands were heavily targeted by boat-based fishers prior to the enforcement of marine park regulations, as the islands are adjacent to the largest town and boating facilities in the region. This offshore reserve was also one of the most commonly patrolled because of its central location and proximity to a relatively safe ocean bar crossing. The substantial reduction in fishing effort combined with the greatest compliance effort would have contributed to the Tollgate Islands reserve showing the strongest positive responses to protection over the first 5 years.

Effective compliance cannot be underestimated in achieving positive marine conservation outcomes [13], [14]. As expected, enforcement actions were positively associated with individual reserve performance in the Batemans Marine Park. As well as active enforcement, the Batemans Marine Park operational plan included priority actions aimed at increasing voluntary compliance. This included local education and awareness activities, programs to improve signage and zone markers as well as proactively restricting potentially harmful activities through permitting. In response to these strategies, we contend that public knowledge of the marine park zoning arrangements improved substantially since the parks establishment. For example, marine park awareness by tourists increased from 47% (n = 203) to 72% (n = 36) from 2008 to 2011 (Eurobodalla Shire Council and NSW Marine Park Authority, unpublished data). Public knowledge and support for marine reserves increases voluntary compliance, which can both improve the effectiveness of marine reserves and reduce the costs of enforcement [54], [55]. Greater consideration of compliance planning during establishment and adaptive management of marine reserve networks can enhance voluntary compliance and improve conservation outcomes [24].

Marine reserve size is generally regarded as a fundamental principle in effective marine reserve design with larger reserves often having greater conservation benefits [56]. By this criterion, the largest marine reserve in our study, Mullimburra, did not perform as well as smaller reserves in the network. Consequently, factors other than reserve size must have been driving this result. Importantly, the Mullimburra reserve had many characteristics considered important for effective reserve design (Table 1). Mullimburra marine reserve was adjacent to the Eurobodalla National Park minimizing potential land-based threats to the marine ecosystem [57], [58]. It was also directly linked to no-take estuarine reserves ensuring undisrupted connectivity between juvenile and adult habitats [59], [60]. Mullimburra marine reserve had cross-shelf coverage from the shore to the 3 nm limit of NSW state waters, maximizing reef habitat representation [61], which is known to be extensive in inner- and mid-shelf waters in the region [51]. It was also surrounded by extensive partially-protected areas (i.e. habitat protection zones) buffering it from unintentional commercial fishing activities [62].

Given all these key reserve attributes, it is not clear why the large reserve at Mullimburra did not perform as well as some smaller reserves, although it should be noted that the BRUV sites in fished areas adjacent to this reserve were further away than they were for other reserves. A more likely explanation is the influence of compliance levels because the least effective reserves, Murramarang and Mullimburra, also had the lowest number of enforcement actions per unit area. A review of compliance related issues from the Great Barrier Reef Marine Park suggests that even a small amount of poaching can have major ecological consequences [26]. Although there are no data available to discriminate between compliance efficacy and the amount of illegal fishing activity in the Batemans Marine Park, the significant relationship between enforcement actions and reserve performance suggests that quantitative monitoring of compliance and illegal activities should be prioritized to facilitate adaptive management to maximize marine conservation outcomes.

It is not possible from our results to determine whether the performance of individual marine reserves within the first 5 years will be a useful predictor of long-term reserve performance. This raises important questions about how much park-specific ecological monitoring can contribute to evidence-based adaptive management of marine park zoning arrangements at a 5 year review, as is currently required in NSW. Certainly, clear advice can be given that the network of marine reserves in the Batemans Marine Park had a positive influence on the abundance of fishes, particularly kyphosids, despite differences in the performance of the individual marine reserves we examined. In contrast, there were no consistent effects to validate the efficacy of habitat protection zones. Ongoing enforcement will also be required to maintain reserve efficacy and extra compliance attention should be focused on the large marine reserves at Mullimburra and Murramarang, which appear to be underperforming given their attributes (see Table 1).

Apart from this general advice, 5 years of ecological monitoring was insufficient to provide scientific evidence that would justify changing the current network of marine reserves and partially-protected areas in the Batemans Marine Park to improve long-term conservation of biodiversity. Nonetheless, the broader scientific literature about marine reserves will still have an invaluable contribution to the review process, with rigorous assessments of reserve attributes (e.g. size, habitat linkages, buffering) from much older marine reserve networks being particularly informative. After the initial 5 year review, NSW marine parks are reviewed every 10 years. At the 15 year review, sufficient time should have passed for substantial changes in the structure of marine communities to have occurred [28], [30]. At this point, the results from local ecological monitoring and other complimentary research will be in a much stronger position to drive evidence-based adaptive management to enhance long-term conservation objectives.

In conclusion, few studies have examined changes in fish assemblages across a network of marine reserves relative to fished areas with different levels of environmental protection. We show that after 5 years of protection, fish abundances were 37% greater across the network of marine reserves compared to partially-protected areas, although not all individual reserves performed equally and performance was temporally variable. These changes are relatively modest compared to some reserve networks (e.g. [63]), but still add to the growing weight of evidence that conservation outcomes from planned networks of marine reserves are greater than those from individual reserves [4], [26]. Our results also provide insight into factors (e.g. past fishing effort and compliance) that promote early conservation benefits to fish in temperate marine reserves and thus should be carefully considered in marine reserve establishment and management. As coastal population growth and associated development increases stress on marine environments, it is critical that networks of marine reserves are designed and adaptively managed to maximise their conservation objectives. Although local environmental monitoring can contribute to adaptive management of newly established marine reserve networks, the extent of this contribution will be limited by the rate of change in marine communities in response to protection. The adaptive management processes of newly established marine reserve networks could, therefore, be enhanced by rigorous assessment of the efficacy of ecological attributes and planning principles from much older networks.
